# Treatment of neonatal dural arteriovenous fistula with a spiral coil combined with glue

**DOI:** 10.1007/s13760-023-02397-6

**Published:** 2023-10-05

**Authors:** Jianbing Ren, Juan Chen, Yongxi Liu, Xiaopeng Zhao, Chuan Nie

**Affiliations:** 1 National Key Clinical Specialty Construction Project/Neonatology Department, Guangdong Women and Children Hospital, Guangdong Neonatal ICU Medical Quality Control Center, No. 521, Xingnan Avenue, Panyu District, Guangzhou, 510000 Guangdong China; 2 Radiology Department, Guangdong Women and Children Hospital, Guangzhou, China; 3 Neonatology Department, Guangzhou Women and Children’s Medical Center, Guangzhou, China

**Keywords:** Dural arteriovenous fistula, Neonatal, Embolism, Endovascular treatment

Dear Editor,

Dural arteriovenous fistulas (DAVFs) are abnormal shunts between the meningeal arteries and dural venous sinuses or cerebral veins and are commonly treated using interventional embolization. Recently, we encountered a rare and interesting case of DAVF in a newborn, and herein we describe imaging manifestations of the condition and clinical treatment of the patient.

One hour after birth, an infant delivered by cesarean section at full term, with a birthweight of 3.16 kg, no history of resuscitation for asphyxia, and no abnormal antenatal findings, was admitted with dyspnea and positive triple concave signs. Dyspnea persisted despite nasal continuous positive airway pressure ventilation. Laboratory examination revealed no apparent abnormality. Cardiac-enhanced computed tomography depicted a widened jugular vein and enlarged right atrium (Fig. 1a). Magnetic resonance angiography and venography revealed a massive DAVF near the right sinus confluence and a small right vertebral artery (Fig. 1b).

**Fig. 1:**
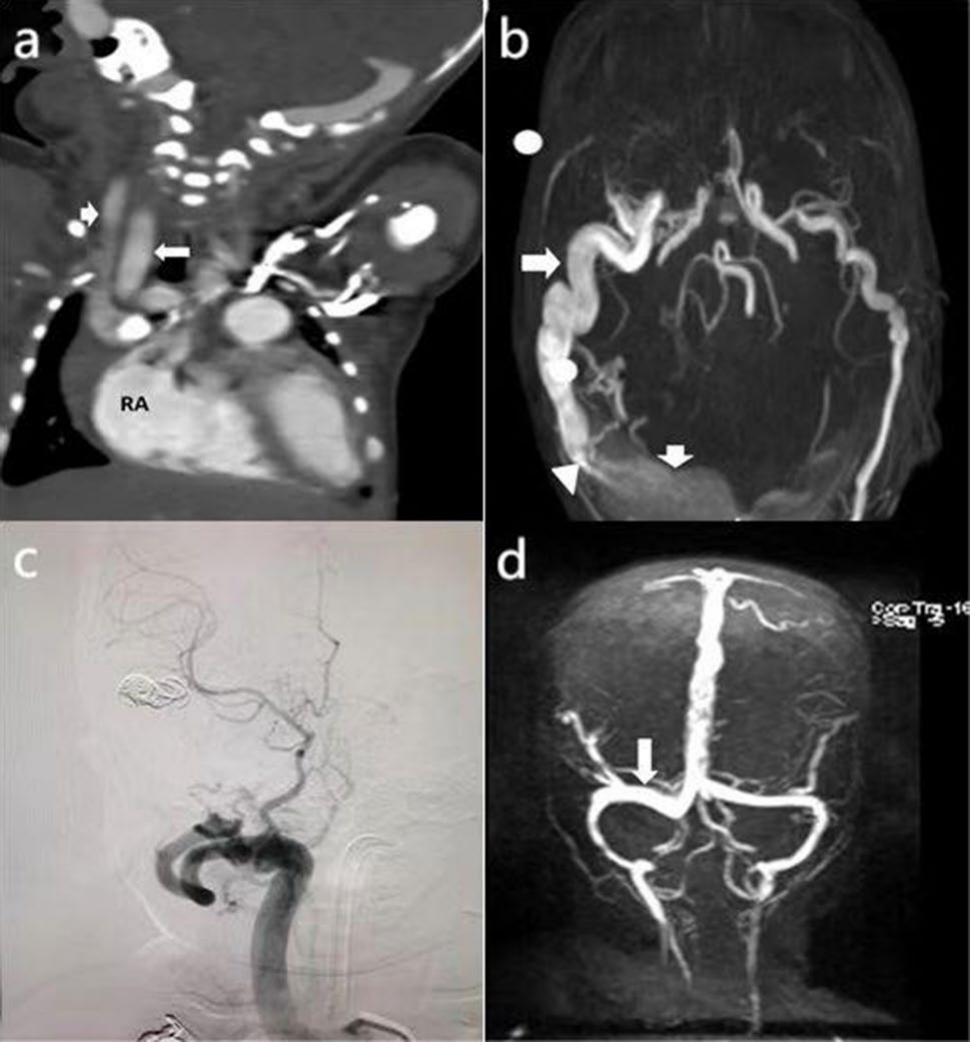
**a** Computed tomography: enhanced coronal view of the heart. The right carotid artery and jugular vein are dilated, and the right atrium is enlarged. Long arrow, right external carotid artery; short arrow, right external jugular vein; *RA* right atrium. **b** Head magnetic resonance angiography. The dilated right dural artery and dilated venous sinus form an arteriovenous fistula. Long arrow, right dural artery; short arrow, right transverse sinus; triangular arrow, arteriovenous fistula. **c** Digital subtraction angiography after the first embolization. Abnormal shunts, such as the arteriovenous fistula between the right dural artery and the dilated venous sinus, disappeared after embolization. **d** Re-examination 4 months after the second operation. Magnetic resonance venography shows that the dilated right transverse sinus has become normal. Long arrow, right transverse sinus

After obtaining written informed consent from the parents, a neurosurgeon performed aortic arch angiography, total cerebral angiography, and interventional embolization of the DAVF in the right lateral sinus under general anesthesia. Intraoperatively, the main arterial blood supply of the fistula was found to be a grossly dilated circuitous branch of the right middle meningeal artery, with collection from the other two central meningeal artery branches. The first branch of the left external carotid artery also supplied blood to the fistula, but the left intracranial vessels were developed, and "blood theft" was not serious; therefore, this was not initially treated. Interventional embolization surgery was planned in consideration of the infant's tolerance for surgery and the limitations of contrast medium and ionizing radiation.

The surgical team performed the first-stage operation 15 days after birth. Considering that the main blood supply artery of the fistula is a branch of the right middle meningeal artery, the diameter of the fistula was obviously dilated, and the shape was highly tortuous. The surgeons packed sequentially six spiral coils to stabilize the fistula until blood flow from the right middle meningeal artery stopped (Fig. 1c). One month later, the surgical team performed the second operation according to the original plan and the results of the first operation were re-evaluated. They found that the external carotid artery branches of the last embolization were thickened as before, but venous sinus dilatation had improved significantly since the previous operation. The surgical team superselected the right middle meningeal artery again and four spiral coils were stabilized at the fistula. The blood supply in the other displayed that the image of the middle meningeal artery was not severe, and the shape was circuitous and far. The surgeon injected 20% Glubran 2 surgical glue (GEM, Viareggio, Italy). Re-examination showed that embolization was satisfactory. During the 10-month follow-up period, the infant had normal head circumference and did not present hydrocephalus, while nerves and behavior developed normally (Fig. 1d).

DAVF is caused by venous sinus wall hyperplasia and abnormal sigmoid sinus, transverse sinus, and sinus confluence development. Perinatal mortality from associated cardiopulmonary and cerebrovascular complications is high. A typical sign of congenital DAVF is a vascular murmur in the head coincident with the heartbeat, but neonatal incidence is rare, and this sign is easily missed. The patient had four branches of the middle dural artery supplying blood to the fistula. Although Borden's classification was type I, the patient had symptoms of dyspnea and right heart failure after birth, consistent with intraoperative observations. Given the multiple intracranial vascular malformations, patient tolerance, and effects of contrast medium and ionizing radiation, we performed numerous embolization procedures using spirals combined with 20% Gluran 2 surgical glue, successfully reducing blood flow in the affected area. This case is one of a few in which DAVF was successfully embolized at the neonatal period using a spiral coil combined with glue [1, 2]. This condition is rare but severe and warrants increased clinical attention. Due to the various clinical manifestations of DAVF, symptoms are related to the number and anatomical location of the fistula, cortical venous drainage, and degree of venous sinus occlusion. Clinicians need to develop highly personalized treatment strategies to address complex DAVF cases.

## Data Availability

All data generated or analyzed during this study are included in this published article.
